# Adverse childhood experience referring to parental relationship is associated with the risk of alcohol dependence and with COMT Val158Met polymorphism, but out of gene-environment interactions

**DOI:** 10.1192/j.eurpsy.2022.348

**Published:** 2022-09-01

**Authors:** T. Merkulova, N. Chuprova, M. Solovieva, A. Nikolishin, A. Kibitov, S. Grechany, N. Baranok, K. Rybakova, V. Soldatkin, A. Yakovlev, A. Trusova, P. Ponizovsky, E. Krupitsky

**Affiliations:** 1 Serbsky National Medical Research Center on Psychiatry and Addictions, Laboratory Of Molecular Genetics, Moscow, Russian Federation; 2 Saint Petersburg State Pediatric Medical University, Department Of Psychiatry And Addictology, Saint Petersburg, Russian Federation; 3 Murmansk Regional Addiction Hospital, Rehabilitation Center, Murmansk, Russian Federation; 4 Bekhterev National Medical Research Center for Psychiatry and Neurology, Department Of Addictology, Saint Petersburg, Russian Federation; 5 Rostov State Medical University, Department Of Psychiatry, Addictology And Medical Psychology, Rostov-on-Don, Russian Federation; 6 Lipetsk Regional Addiction Hospital, Department Of Addictology, Lipetsk, Russian Federation; 7 Saint Petersburg State University, Department Of Medical Psychology And Psychophysiology, Saint Petersburg, Russian Federation; 8 Serbsky National Medical Research Center on Psychiatry and Addictions, Department Of Therapy Of Mental Disorders Complicated By Addiction Diseases, Moscow, Russian Federation

**Keywords:** Alcohol dependence, GxE interaction

## Abstract

**Introduction:**

Gene-environment interactions (GxE) are considered to make a substantial impact on the risk of alcohol dependence (AD).

**Objectives:**

The aim of the study: to test the associations between the functional polymorphism Val158Met (rs6265) in the catechol-O-methyl transferase (COMT) gene, affecting dopamine neurotransmission, and adverse childhood experiences (ACE) and their GxE interactions with AD risk.

**Methods:**

The study included 149 AD inpatients (mean age 29.9 (SD=3.91), 16.1% females) and 201 healthy volunteers (23.3 (2.48), 30.1% females). The Adverse Childhood Experiences International Questionnaire (ACE-IQ) was used for assessing ACE. COMT Val158Met polymorphism was detected by RT-PCR.

**Results:**

First, COMT Val158Met polymorphism was associated only with adverse childhood experience referring o parental relationship (ACE-IQ), but differently in two groups. Healthy minor Met158 carriers have lower scores on the subscale “relationship with parents/guardians” (P) (p=0.025) and “physical neglect” (PN) (p=0.059) vs. homozygous Val158 carriers. However, AD patients - Met158carriers have a tendency to a higher score on the subscale “one or no parents, parental separation or divorce” (PSD) (p=0.078). Then logistic regression revealed associations of these ACE scores with increased AD risk: P (p=0.001, OR=1.186, 95%CI [1.069-1.315]), PN (p=0.024, OR=1.254, 95%CI [1.030-1.526]), and PSD (p=0.016, OR=1.499, 95%CI [1.080-2.082]). No associations of COMT Val158Met alone or in interactions with these ACE-IQ scores with the AD risk were found.

**Conclusions:**

Adverse childhood experience referring to parental relationship is associated with alcohol dependence risk and separately with COMT Val158Met, but no clear interactions in frame of GxE has been supported.

**Disclosure:**

No significant relationships.

